# Signal peptides for recombinant protein secretion in bacterial expression systems

**DOI:** 10.1186/s12934-018-0901-3

**Published:** 2018-03-29

**Authors:** Roland Freudl

**Affiliations:** 1 Institut für Bio- und Geowissenschaften 1, Biotechnologie, Forschungszentrum Jülich GmbH, 52425 Jülich, Germany; 2 Bioeconomy Science Center (BioSC), Forschungszentrum Jülich GmbH, 52425 Jülich, Germany

**Keywords:** Protein secretion, Recombinant protein production, Signal peptide, Sec pathway, Twin-arginine-translocation (Tat) pathway, Gram-positive bacteria

## Abstract

The secretion of biotechnologically or pharmaceutically relevant recombinant proteins into the culture supernatant of a bacterial expression host greatly facilitates their downstream processing and significantly reduces the production costs. The first step during the secretion of a desired target protein into the growth medium is its transport across the cytoplasmic membrane. In bacteria, two major export pathways, the general secretion or Sec pathway and the twin-arginine translocation or Tat pathway, exist for the transport of proteins across the plasma membrane. The routing into one of these alternative protein export systems requires the fusion of a Sec- or Tat-specific signal peptide to the amino-terminal end of the desired target protein. Since signal peptides, besides being required for the targeting to and membrane translocation by the respective protein translocases, also have additional influences on the biosynthesis, the folding kinetics, and the stability of the respective target proteins, it is not possible so far to predict in advance which signal peptide will perform best in the context of a given target protein and a given bacterial expression host. As outlined in this review, the most promising way to find the optimal signal peptide for a desired protein is to screen the largest possible diversity of signal peptides, either generated by signal peptide variation using large signal peptide libraries or, alternatively, by optimization of a given signal peptide using site-directed or random mutagenesis strategies.

## Background

Recombinant proteins, such as technical bulk enzymes and biopharmaceutical proteins, represent a multi billion dollar market. For the biotechnological production of these proteins, different pro- and eukaryotic expression systems are currently used [1]. Among those, bacteria are especially interesting as expression hosts since they are comparably easy to handle and, in many cases, a multitude of tools exist for their genetic manipulation [2].

The secretion of recombinant proteins into the growth medium of the respective bacterial host organisms possesses several important benefits compared to intracellular expression strategies. First, secretion of aggregation prone target proteins can prevent their accumulation as insoluble inclusion bodies in the cytosol. Second, the toxic effect exerted by some target proteins on the production host upon their intracellular expression can be reduced or even be alleviated when the respective protein is secreted out of the cell into the surrounding culture medium. Third, since many interesting target proteins (such as, e.g. therapeutic antibodies) require the correct formation of disulfide bonds for their final conformations and biological activities, the secretion of the respective proteins into an extracytoplasmic compartment is an essential step for their production since disulfide bond formation is effectively prevented in the reducing environment of the cytosol. Finally and most importantly, the secretion of a desired target protein into the growth medium greatly simplifies product recovery, since no cell disruption is required and the subsequent purification and downstream processing steps can be significantly reduced. Due to this, the secretory production of a given target protein can drastically decrease the overall production costs [3].

Gram-positive bacteria usually possess only a single membrane (i.e. the cytoplasmic membrane) and export of a target protein across this major permeability barrier can directly result in its release into the culture supernatant. Due to this fact, members of this class of microorganisms are considered especially useful as potential host organisms for the secretory production of industrially relevant recombinant proteins. In fact, since many years various Gram-positive bacteria (e.g. various *Bacillus* species) are extensively used in industry for the secretory production of a variety of technical enzymes such as lipases, amylases, and proteases, resulting in product yields of more than 20 g/l in the respective culture supernatants [4]. However, these exceptional high product yields are obtained predominantly only for naturally secreted enzymes that originate either directly from the production host itself or from one of its close relatives. In contrast, the yields obtained for heterologous proteins are often comparably very low or the desired target proteins were not secreted at all [5, 6]. Due to this fact, it is important to test different alternative secretory expression systems such as, e.g. *Lactococcus lactis* [7], *Streptomyces lividans* [8], or *Corynebacterium glutamicum* [9] for their ability to express and secrete heterologous proteins that cannot efficiently be produced by established host organisms, such as the commonly used Gram-positive model bacterium *Bacillus subtilis* [10]. As outlined further below in more detail, one of the most critical parameters that are decisive whether an attempt to secrete a desired target protein becomes successful and/or economically relevant or not is the nature of the signal peptide that is used to transport the protein across the cytoplasmic membrane of the respective host microorganism.

## Two major bacterial protein export pathways: Sec and Tat

**Fig. 1:**
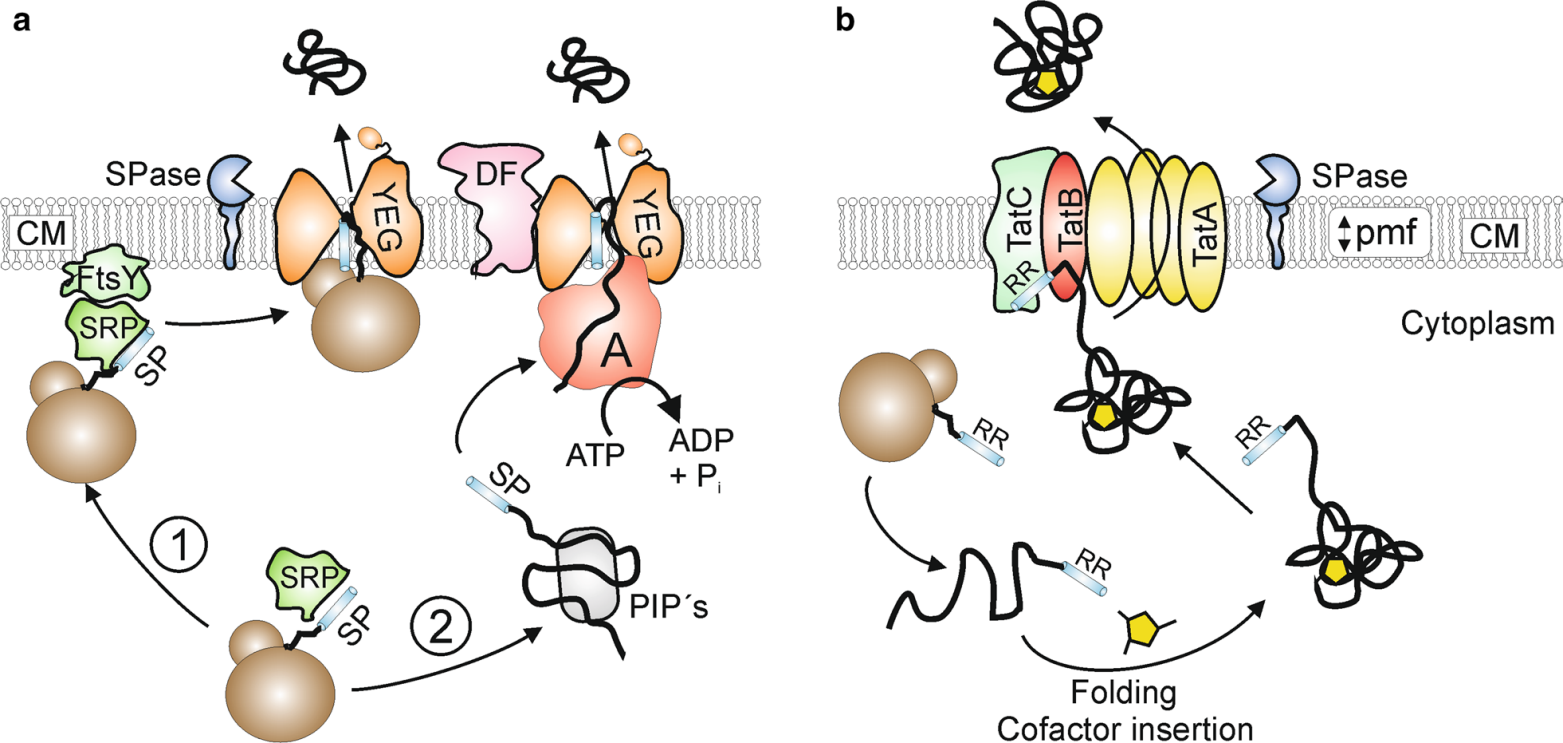
Two major bacterial export pathways: Sec and Tat. **a** The general secretion (Sec) protein export pathway. In the cotranslational mode (1), Sec substrates possessing highly hydrophobic signal peptides (SP) are recognized at the ribosome by the signal recognition particle (SRP). Subsequently, the ribosome-nascent chain (RNC)-SRP complex docks to the SRP-receptor FtsY and the RNC is then further transferred to the SecYEG translocation pore such that ribosomal exit site is in close proximity to SecYEG. The energy for translocation in the cotranslational export mode is provided by further elongation of the substrate at the ribosome. In the posttranslational mode (2), Sec-dependent precursor proteins are kept in an export-competent state by posttranslationally interacting proteins (PIP’s) such as SecB, the general chaperones GroELS/DnaK–DnaJ–GrpE or the soluble form of SecA. The signal peptide (SP) is recognized by the SecA protein which pushes the protein through the SecYEG protein conducting channel in a stepwise and ATP-dependent manner. In addition, SecDF exerts a proton motive force (pmf)-dependent pulling force on the substrate from the trans-side of the cytoplasmic membrane (CM). During or shortly after translocation, the signal peptide is removed by signal peptidase (SPase) and the mature protein is released on the trans-side of the CM. **b** The twin-arginine translocation (Tat) protein export pathway. After folding and, if required (as shown here), cofactor insertion, preproteins containing a signal peptide with a twin-arginine motif (RR) are recognized by a receptor complex consisting of TatC and TatB. Subsequently, homooligomeric complexes of TatA are recruited to the substrate-loaded receptor complex in a proton motive force (pmf)-dependent manner, followed by the translocation of the substrate across the cytoplasmic membrane (CM). Following to substrate translocation, the signal peptide is cleaved by signal peptidase (SPase) and the mature protein is released on the trans-side of the membrane

In bacteria, the ubiquitous general secretion (or Sec) pathway (Fig. 1a) is the most important transport route for proteins that are exported out of the cytosol [11]. In most cases, Sec substrates are synthesized as higher molecular weight precursor proteins possessing an amino-terminal signal peptide that is responsible for the targeting of the respective proteins to the membrane-bound Sec translocase [12]. The actual membrane translocation of exported proteins via the Sec pathway takes place in an unfolded state and can occur either in a cotranslational or in a posttranslational manner [13]. In the cotranslational export mode, precursor proteins possessing a highly hydrophobic signal peptide are recognized by a signal recognition particle (SRP) already during their synthesis at the ribosome. Subsequently, the ribosome-nascent chain complex (RNC) is targeted to the membrane-associated SRP receptor (FtsY), followed by transfer of the RNC complex to the actual translocation pore that consists of the integral membrane proteins SecYEG. Upon binding of the RNC to the cytoplasmic side of the SecYEG channel, the exported protein is directly synthesized by the ribosome into the translocation pore [14]. In the posttranslational mode, precursor proteins possessing less hydrophobic signal peptides are fully synthesized and, upon release from the ribosome, interact with posttranslationally interacting proteins (PiP’s), such as the specific targeting chaperone SecB [15], the general chaperones GroEL–GroES [16], DnaK–DnaJ–GrpE [17], or the soluble form of SecA [18], which protect the export proteins from aggregation and maintain them in an unfolded, export competent state. The precursor proteins are then delivered to the translocase and pushed through the SecYEG pore in a stepwise fashion by the translocation ATPase component SecA [19]. Additionally, a SecYEG-associated complex consisting of SecD and SecF also exerts a pulling force on the translocating polypeptide chain from the extracytosolic side of the membrane [20]. During or shortly after the membrane translocation event, the signal peptide is removed by signal peptidase and the mature protein is released on the trans-side of the membrane [21].

In many (but not all) bacteria, another major protein export pathway exists that remarkably transports its substrates in a completely folded or even oligomeric state (Fig. 1b). This alternative export pathway has been named the twin-arginine translocation (or Tat) pathway due to the presence of a twin-arginine pair that is present in the signal peptides of the respective Tat substrates [22]. The Tat translocase consists of the components TatA, TatB, and TatC in Gram-negative bacteria and Gram-positive bacteria with a high GC-content, whereas a minimal translocase consisting solely of TatA and TatC is operating in Gram-positive bacteria with a low GC-content [23–25]. In the latter case, the TatA protein is bifunctional and, besides the TatA functions, also takes over the role of TatB [26, 27]. Following their synthesis and cytoplasmic folding which, in many cases includes the insertion of a tightly or even covalently bound cofactor, the fully folded precursor proteins bind to a substrate receptor in the cytoplasmic membrane which is formed by TatB (or alternatively by a bifunctional TatA protein) and TatC [28, 29]. Subsequently, multimers of TatA are recruited in a protonmotive-force-dependent manner to the substrate-loaded receptor complex [30], upon which the substrate is translocated either through a size-fitted pore consisting of a variable number of TatA molecules [31] or, alternatively, through a weakened patch of the membrane near the substrate receptor complex that is induced by TatA [32]. Finally, also in the Tat pathway, the signal peptide is removed by signal peptidase and the mature protein is released on the trans-side of the membrane [33].

## Sec and Tat signal peptides: general features and prediction programs

**Fig. 2:**
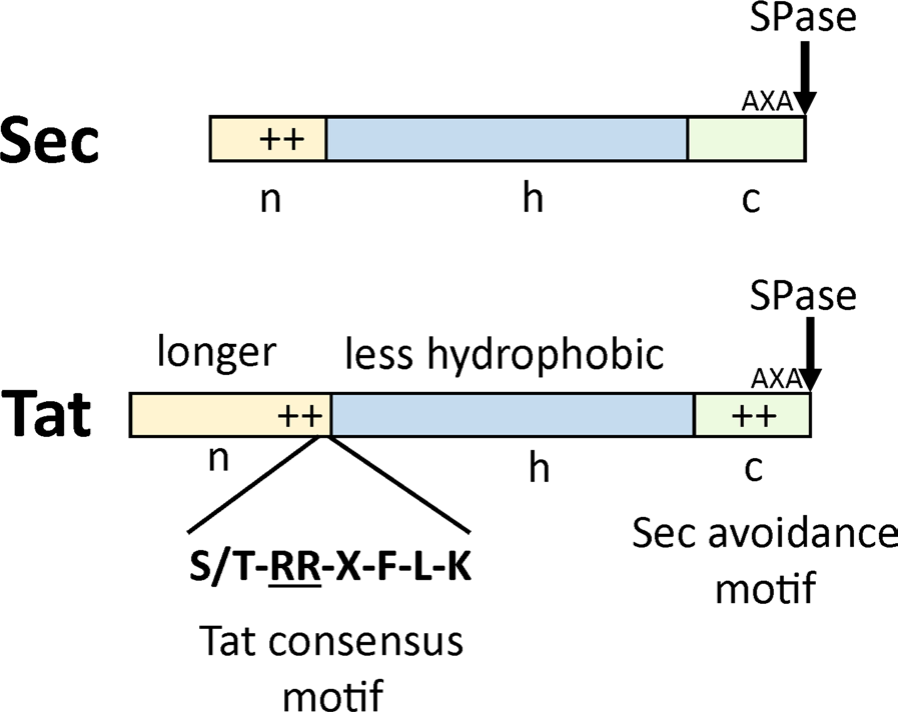
General features of Sec and Tat signal peptides. Sec and Tat signal peptides possess a similar tripartite overall structure consisting of a positively charged n-region, a central hydrophobic h-region, and a polar c-region that contains the recognition site (consensus: A-X-A) for signal peptidase (SPase; the cleavage site is indicated by an arrow). In Tat signal peptides, a characteristic amino acid consensus motif including two highly conserved arginine residues (underlined) is present at the boundary between the often significantly longer n-region and the h-region. Furthermore, the h-region of Tat signal peptides is mostly less hydrophobic than those found in Sec signal peptides and in the c-region of Tat signal peptides, frequently positively charged amino acids (the so-called Sec-avoidance motif) are present that prevent a mistargeting of Tat substrates into the Sec pathway

Signal peptides are short amino-terminal parts of exported precursor proteins that direct the respective proteins to the protein export systems in the cytoplasmic membrane. Sec signal peptides generally do not show sequence similarities, however a conserved tripartite overall structure can be recognized (Fig. 2, upper part), which consists of a positively-charged amino-terminal n-region, a central hydrophobic core (h-region), and a polar carboxyl-terminal domain (c-region) that contains the signal peptidase recognition site (consensus motif A-X-A) [12]. All three domains contribute to the efficient export of Sec substrates in vivo. Changing the positive net charge of the n-region to zero or a negative value significantly reduces the transport rate [34]. Disrupting the hydrophobic core by a polar or charged amino acid residue also decreases or even completely abolishes membrane transport, indicating that a minimal length and a minimum hydrophobic density of the h-region is required to promote membrane translocation [35, 36]. Finally, as a prerequisite for correct cleavage of the signal peptide by signal peptidase, amino acids harboring small neutral side chains must occupy the − 3 and the − 1 position in the c-region [37, 38].

Tat signal peptides possess a similar tripartite overall structure as Sec signal peptides, likewise consisting of an n-, h-, and c-region, respectively (Fig. 2, lower part). Nevertheless, several important differences have been noticed that are involved in maintaining the export pathway specificity of Tat substrates. First, a conserved amino acid consensus motif (S/T-*R*-*R*-X-F-L-K, where X often is a polar amino acid residue) is present at the boundary between the often comparably longer n-region and the h-region of Tat-specific signal peptides [39] and it has been demonstrated in numerous studies that this Tat consensus motif is a major determinant for the specific binding of Tat precursor proteins to the Tat translocase [22]. Second, the h-region of Tat signal peptides is mostly less hydrophobic than those found in Sec signal peptides [40]. Third, in the c-region of Tat signal peptides, sometimes positively charged amino acids can be found that rarely are present in the c-region of Sec signal peptides which, together with the lower hydrophobicity of the h-region, prevent a faulty interaction of Tat substrates with the Sec export machinery (“Sec avoidance”) [40, 41].

For the prediction of signal peptides, several bioinformatic tools have been developed that are either based on weight matrices, sequence alignments, neural networks, or machine learning algorithms, respectively [42]. For Sec signal peptides, Phobius (http://phobius.sbc.su.se/ [43]), Philius (http://www.yeastrc.org/philius/pages/philius/runPhilius.jsp [44]), and SignalP (current version 4.1; http://www.cbs.dtu.dk/services/SignalP [45]) are among the top scoring and most popular analysis programs that predict the likelyhood of a given amino acid sequence for being a Sec signal peptide or not. A discrimination score (D-score) is assigned by SignalP to the analyzed peptides and sequences with a D-score higher than 0.5 are classified as putative signal peptides, whereby sequences possessing a D-score above 0.7 having a high probability that they in fact really do so [45].

For the identification of Tat signal peptides, TatP (http://www.cbs.dtu.dk/services/TatP/ [46]), TatFind (http://signalfind.org/tatfind.html [47]), and PRED-Tat (http://www.compgen.org/tools/PRED-TAT/ [48]) are commonly used prediction programs. However, since twin-arginine residues can also occur in Sec signal peptides, these programs tend somewhat to overestimate the number of Tat substrates in a given organism. Due to this, an experimental verification that a putative Tat substrate in fact uses the Tat pathway for its export is still of crucial importance. Nevertheless, the mentioned signal peptide prediction programs represent a valuable tool to scan the genomes of different organisms for signal peptides that subsequently can be tested with respect to their performance in the secretion of a desired heterologous target protein by a given bacterial expression host.

## Steps in the secretory protein production process that are affected by signal peptides

As mentioned above, signal peptides discriminate exported proteins from proteins that remain in the cytosol. Signal peptides mediate the targeting and binding of exported precursor proteins to the respective protein translocases in the cytoplasmic membrane [49]. An additional important role of Sec signal peptides that mediate a posttranslational mode of export is to slow down the folding of the attached mature protein part to allow its efficient interaction with posttranslationally interacting proteins (such as SecB) and, by this means, help to maintain the respective export proteins in their export competent state [50, 51]. Furthermore, the gene regions for Sec signal peptides have a strong bias for non-optimal codons, a feature that by slowing down the kinetics of translation has a profound positive effect on the export efficiency and the overall productivity of the secretory production process [52]. Replacing the non-optimal codons by optimal codons in the gene regions for the signal peptides of the *Escherichia coli* maltose-binding protein [53] or β-lactamase [54] resulted in lower protein production which could be partially increased in strains that are defective in multiple proteases or at lowered temperatures. This indicates that slowing down the rate of translation by means of the rare codons present in Sec signal peptides is highly important to ensure an efficient interaction of the export proteins with the components of the export machinery and to prevent their degradation. Additionally, Sec signal peptides have also been found to function as allosteric activators of the Sec translocase [55].

Besides these steps in the secretory protein production pathway that directly determine the efficiency and kinetics by which a protein is targeted to and translocated across the cytoplasmic membrane, signal peptides also indirectly have an effect on the overall production process. For example, the fusion of different signal peptides to a given target protein results in different mRNA transcripts that can vary in their secondary structure and/or in their stability and, due to this, significantly can influence the amounts of the respective precursor proteins that are synthesized [56, 57].

## Signal peptide variation and modification: powerful tools for optimizing heterologous protein secretion

As outlined in the previous chapter, signal peptides are not just simple address codes for exported proteins, but rather affect various stages of the entire secretory protein production process. Due to this, it is obvious that the nature of the signal peptide that is used for the secretion of a desired target protein is of crucial importance with respect to the final yield of the respective protein in the culture supernatant of a microbial host organism and that finding the best possible signal peptide for a given target protein is one of the most critical steps on the way to an efficient secretory production process.

### Signal peptide variation

**Fig. 3:**
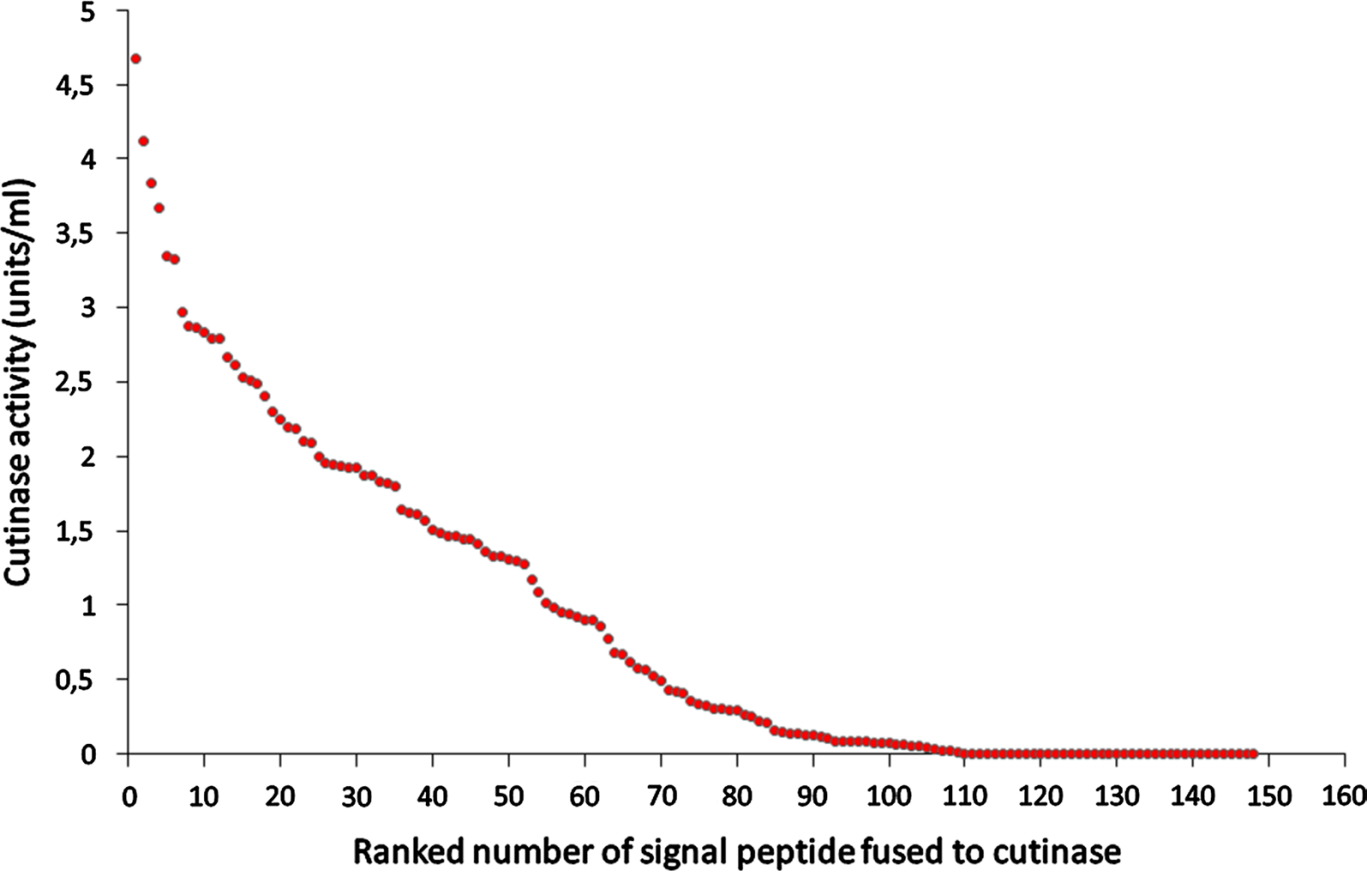
Signal peptide variation. Influence of 148 different Sec signal peptides from *B. subtilis* on the secretion of the heterologous target protein cutinase from *F. solani pisi* into the culture supernatant of *B. subtilis*. The signal peptides are ranked and numbered according to their performance in cutinase secretion as reported by Brockmeier et al. [58]

More than a decade ago, the first systematic study on the effects of signal peptide variation on the secretory production of heterologous proteins was reported by Brockmeier et al. [58]. In this study, 173 predicted Sec signal peptides from *B. subtilis* were individually fused in an identical manner to a cutinase from the fungus *Fusarium solani pisi*. The genes for the resulting hybrid precursor proteins were subsequently expressed in *B. subtilis* and the resulting enzyme activities in the culture supernatants of the individual clones were determined for each signal peptide-target protein combination. As shown in Fig. 3, the enzymatic activities obtained in the culture medium of the recombinant *B. subtilis* cells varied significantly depending on the nature of the signal peptide that was used to direct the cutinase into the Sec export pathway. Whereas with the best signal peptide (derived from the secreted protease Epr) 4.67 units/ml of cutinase activity could be obtained, a significant number (i.e. 39) of signal peptides did not even resulted in the secretion of detectable cutinase activity. Importantly, no correlation between the performances of the different signal peptides in cutinase secretion and their D-scores predicted by the SignalP program could be detected. Strikingly, when another unrelated heterologous target protein (an esterase of metagenomic origin) was analyzed in an identical manner using a selected set of signal peptides, it became clear that the best signal peptide for cutinase secretion was a poor signal peptide for the secretion of the esterase. Vice versa, the best signal peptide for esterase secretion was only a mediocre signal peptide for cutinase. These results clearly demonstrated that for each individual target protein an optimally fitted signal peptide has to be identified [58].

Identical findings were obtained when a signal peptide library consisting of 76 Sec signal peptides was analyzed for their performance in the secretory production of a staphylococcal nuclease (NucA) with *Lactobacillus plantarum* WCSFI as expression host [59]. Also in this case, the amounts of secreted NucA varied drastically depending on the signal peptide. Furthermore, when a subset of the signal peptides was analyzed with respect to the secretion of an unrelated lactobacillal amylase (AmyA), no correlation was found between signal peptide performance with NucA and the corresponding signal peptide performance with AmyA [59].

An even larger signal peptide library consisting of 173 signal peptides from *B. subtilis* and 220 signal peptides from *Bacillus licheniformis* was used in a high throughput screening approach to find the optimal signal peptide for the secretion of the *Bacillus amyloliquefaciens* protease subtilisin BPN’ in three different expression host organisms (*B. subtilis* TEB1030, the *B. licheniformis* type strain DSM13/MW3, and the industrially relevant *B. licheniformis* strain H402). Also in this study, a strong dependence of the amounts of secreted subtilis BPN’ from the signal peptide used was noticed. Interestingly, a similar relative performance of the great majority of the signal peptides in the secretion of subtilisin BPN’ was observed in all three *Bacillus* expression strains [60].

In another study, 405 Sec and Tat signal peptides predicted from the genome of *C. glutamicum* R were individually tested for their performance in the secretion of a heterologous α-amylase from *Geobacillus thermophilus* by *C. glutamicum* R as the expression host [61]. 108 of these signal peptides (further classified in 98 Sec-type and 10 Tat-type signal peptides) mediated detectable export of the α-amylase and, also in this study, drastic differences in the amounts of secreted amylase were observed for different signal peptides. Strikingly, the highest α-amylase yield was obtained when the normally Sec-dependent amylase was directed into the Tat pathway by the Tat-specific CGR0949 signal peptide, indicating that switching the protein export pathway can significantly increase the yield of a given target protein and, therefore, can be a very effective additional option for the improvement of secretory protein production processes [61].

Recently, the signal peptide–cutinase fusion library used by Brockmeier et al. [58] for the analysis of cutinase secretion in *B. subtilis* was transferred to the distantly related expression host *C. glutamicum* ATCC13032 [62]. In contrast to the above-mentioned findings of Degering et al. [60] which used three closely related *Bacillus* strains as host organisms, the relative performance of the signal peptides with respect to cutinase secretion was found to differ drastically when compared between *B. subtilis* and *C. glutamicum*, indicating that the optimal fitted signal peptide for a given target protein has to be identified from scratch for each expression host [62].

The power of using large signal peptide libraries for the optimization of heterologous protein secretion has been reported also in several additional studies, e.g. [63–65], and meanwhile, a “*Bacillus subtilis* Secretory Expression System” that is based on the signal peptide library described by Brockmeier et al. [58] has even been commercialized by TaKaRa/Clontech (http://www.clontech.com/CU/Products/Protein_Expression_and_Purification/Bacterial_Expression_Systems/High_Yield_Expression/B_subtilis_Secretory_Protein).

Besides the use of large signal peptide libraries, there are also numerous studies in the literature, too numerous to be listed in their entirety, where small numbers of different signal peptides were tested for their performance in the secretion of heterologous proteins via the Sec, e.g. [66], or the Tat, e.g. [67], pathway. Also in these studies, an enormous influence of the nature of the signal peptide on the final yields of the investigated target proteins has been observed.

### Signal peptide modification

Besides switching entire signal peptides, the modification of a given signal peptide by random or site-directed mutagenesis approaches represent alternative possibilities to improve the secretion of a desired heterologous target protein into the supernatant of a chosen production host and a few selected examples of these alternative strategies will be described in the following section.

The positively charged amino acid residues in the n-region of Sec signal peptides are known to contribute to the export efficiency by which a Sec-dependent precursor protein is translocated across the cytoplasmic membrane [34]. In line with this notion, the yields of mouse tumor necrosis factor secreted by *S. lividans* could be increased sevenfold by increasing the net charge of the n-region of the signal peptide from a *Streptomyces venezuelae* α-amylase from + 2 to + 3 [68]. In contrast, a decrease of the positive net charge of the n-region of the *Streptomyces tendae* α-amylase inhibitor tendamistat signal peptide from + 3 to + 2 resulted in the doubling of the amounts of tendamistat in the supernatant of the heterologous expression host *S. lividans*, whereas increasing the positive net charge to + 4 or + 6 had an adverse effect [69]. Similarly, a saturation mutagenesis of the positions 2–7 of the n-region of the *B. subtilis* α-amylase AmyE signal peptide revealed that three out of four isolated mutant signal peptides that significantly increased the amounts of the heterologous target protein cutinase from *F. solani pisi* in the *B. subtilis* culture supernatant likewise led to a reduction of the net charge of the n-region from + 3 to + 2 [70]. The analysis of the export kinetics of the respective precursor proteins by pulse-chase experiments interestingly revealed that some of the mutated signal peptides actually slowed down the translocation of the cutinase across the cytoplasmic membrane. It was speculated that a highly efficient targeting and translocation of the cutinase might result in an overloading of the extracytosolic folding catalyst PrsA [71], thereby leading to the accumulation of misfolded cutinase on the trans-side of the membrane and to the subsequent induction of cell wall stress-induced proteases (i.e. HtrA and HtrB) which then reduce the overall secreted amount of the cutinase. Such a scenario in fact could explain why, in some cases, decreasing the export rate can lead to a higher amount of a secreted heterologous protein in the culture supernatant of the respective expression host [70]. Altering the net charge of the n-region has been also shown to improve the performance of a Tat-specific signal peptide. Replacement of the lysine at position 38 in the very long n-region of the xylanase signal peptide by a negatively charged glutamate residue resulted in an 219% increase of xylanase secretion by *S. lividans*. Strikingly, also a replacement of a negatively charged aspartate residue at position 41 by an asparagine residue more than doubled the amounts of the xylanase in the *S. lividans* culture supernatant. It was concluded that the number of positively or negatively charged amino acid residues in the n-region is less important than their distribution in the n-domain of the Tat-specific xylanase signal peptide [72].

Besides the charge composition of the n-region, the length and the hydrophobic density of the central h-region of a signal peptide are also critical features that have strong influences on the efficiency of membrane translocation by both the Sec- [35] and the Tat pathway [73], respectively, and a fine tuning of the h-region likewise can result in an improvement of heterologous protein secretion. For example, increasing the length and the hydrophobicity of the signal peptide from the cell wall protein CWP from *Bacillus brevis* by introducing 4 additional leucine residues into the h-region resulted in a significant (i.e. sevenfold) increase in the secretion of tuna growth hormone into the supernatant of *B. brevis*. When additionally the net charge of the n-region was increased from + 2 to + 4, the secretion of the tuna growth hormone by *B. brevis* could be even further improved, i.e. more than 12-fold compared to the unaltered signal peptide [74]. Similarly, the addition of 3 additional leucine residues to the h-region of the CWP signal peptide in combination with an increase of the net charge of the n-region from + 2 to + 4 increased also the secretion of human interleukin-2 by *B. brevis* more than tenfold [75]. Likewise, increasing the hydrophobicity by replacing a serine residue in the h-region of the signal peptide of the heat-stable enterotoxin II signal peptide by leucine or isoleucine significantly improved the membrane translocation and the periplasmic yield of an hu5D5 antibody heavy chain in *E. coli* [76]. In contrast, in other studies it was found that increasing the hydrophobicity of the h-region in fact did not resulted in an increase, but rather in a reduction of the secretion of a *Staphylococcus aureus* nuclease by *L. lactis* [77] or of the *B. amyloliquefaciens* α-amylase AmyQ by *B. subtilis* [78].

The third part of signal peptides that can influence the efficiency by which heterologous proteins are secreted is the c-region. The c-region contains the signal peptidase recognition site which is characterized by the presence of amino acids with small neutral side chains at the positions − 3 and − 1 relative to the signal peptidase cleavage site (− 3, − 1 rule; preferential consensus motif is A-X-A [37]). In line with this, the substitution of a non-optimal threonine residue at the − 3 position in the lactococcal SP310 signal peptide by an alanine resulted in a 10% increase of the secretion of *S. aureus* nuclease by *L. lactis* [77]. Likewise, the replacement of a serine residue at position − 3 in the signal peptide of a transglutaminase from *Streptomyces hygroscopicus* by an alanine improved the secretion of the transglutaminase in the heterologous expression host *S. lividans* by 10–15% [79].

Besides modifying existing signal peptides, also the de novo design of synthetic signal peptides is an option which can increase the secretion of heterologous proteins by a bacterial host organism. Based on the comparison of several efficient signal peptides from various *Streptomyces* species, Mhiri et al. [80] designed a synthetic signal peptide that contained at each position the amino acid that was found most often at the respective positions in the signal peptides analyzed. When tested with respect to the secretion of an amylase (AmyTO1) from a thermophilic *Streptomyces* sp. in the heterologous expression host *S. lividans*, the artificial synthetic signal peptide performed more than eightfold better in the secretion of the amylase into *S. lividans* culture supernatant than the authentic AmyTO1 signal peptide [80].

## Conclusions

In the past few years, it has become increasingly clear that no universal signal peptide exists that promotes the best possible secretory production of any desired target proteins in any chosen bacterial expression hosts. Rather, an optimally fitted signal peptide has to be identified for every individual target protein in order to allow the best possible secretion into the culture supernatant of the respective chosen secretory production host organism. Due to the fact that signal peptides do not only affect the translocation of the target proteins across the cytoplasmic membrane via either the Sec of the Tat protein export pathways, but also have influences on the biosynthesis of the respective precursor proteins and even on events occurring at the trans-side of the cytoplasmic membrane, it is almost impossible possible to predict in advance which signal peptide will perform best in the context of a given target protein and a given expression host. Therefore, the most promising way to find the optimal signal peptide for a desired protein is to screen the largest possible diversity of signal peptides, either generated by signal peptide variation using large signal peptide libraries or by directed or random signal peptide modifications strategies. In this respect, the development of high throughput screening methods that can handle a large number of biological variants and allow the picking of the raisins out of a large bunch of signal peptides in a fast and reliable manner, such as e.g. the automated microbioreactor platform described by Rohe et al. [81], becomes increasingly important.
